# A retrospective analysis of tidal breathing pulmonary function characteristics in infants and young children with recurrent wheezing

**DOI:** 10.3389/fped.2026.1966843

**Published:** 2026-10-26

**Authors:** Qian Ding, Huahua Tian, Yanmin Wang, Xiaowei Wang, Jiao Chen

**Affiliations:** Department of Pediatric Respiratory Medicine II, Shijiazhuang Maternal and Child Health Hospital, Shijiazhuang, Hebei, China

**Keywords:** expiratory flow limitation, infants, recurrent wheezing, tidal breathing pulmonary function, tPTEF/tE, VPTEF/VE, young children

## Abstract

**Background:**

Recurrent wheezing is common in infants and young children, but objective lung function assessment is limited because conventional spirometry is often not feasible. Tidal breathing pulmonary function testing may help characterize expiratory flow abnormalities in this age group.

**Objective:**

To evaluate tidal breathing pulmonary function characteristics in infants and young children with recurrent wheezing.

**Methods:**

This retrospective study included 178 children who underwent tidal breathing pulmonary function testing from March 2025 to March 2026, including 120 with recurrent wheezing and 58 non-wheezing controls. Core indices, abnormality patterns, multivariable logistic regression, sensitivity analyses, and exploratory ROC analysis were evaluated.

**Results:**

Children with recurrent wheezing had a lower ratio of time to peak tidal expiratory flow to total expiratory time (tPTEF/tE) than non-wheezing controls, with a mean difference of −0.041 and *P* < 0.001. The ratio of volume to peak tidal expiratory flow to total expiratory volume (VPTEF/VE) was also lower in the recurrent wheezing group, with a mean difference of −0.045 and *P* < 0.001. Any tidal breathing pulmonary function abnormality was more frequent in the recurrent wheezing group than in controls, at 75.8% and 27.6%, respectively, *P* < 0.001. In a combined multivariable model including both core indices, higher tPTEF/tE and VPTEF/VE were independently associated with lower odds of recurrent wheezing, with adjusted ORs of 0.746 and 0.746 per 0.01 increase, respectively, both *P* < 0.001. Sensitivity analyses excluding premature infants, pneumonia cases, oxygen therapy, bronchodilator exposure, and lower-quality but analyzable tests showed consistent results. Exploratory ROC analysis showed moderate discriminatory ability, with AUCs of 0.779 for tPTEF/tE and 0.792 for VPTEF/VE.

**Conclusion:**

Infants and young children with recurrent wheezing showed lower tPTEF/tE and VPTEF/VE, suggesting altered tidal expiratory breathing mechanics. These indices may provide supportive objective information for evaluating recurrent wheezing, but they should not be used as standalone diagnostic markers.

## 1 Introduction

Recurrent wheezing is common in infants and young children and is a frequent reason for pediatric respiratory visits, hospitalization, and repeated medication exposure. However, recurrent wheezing in early life is not a single disease entity and may reflect transient viral-associated wheeze, early asthma tendency, or mixed airway inflammation. Recent studies emphasize that preschool wheezing should be characterized by age, confirmed wheeze, and recurrent episodes while recognizing that clinical phenotypes alone may not fully explain future risk or treatment response (1, 2). Evaluation of recurrent wheezing in this age group remains challenging. Although spirometry is widely used in older children and adults, it is difficult to perform reliably in infants and young children because it requires patient cooperation and standardized technique. Consequently, clinicians often rely on symptoms, treatment response, and atopic background when making management decisions (3, 4). This approach is clinically useful but does not directly quantify early airway functional impairment.

Early-life lung function has therefore received increasing attention because impaired airway function in childhood may be associated with persistent wheeze, asthma, and less favorable lung-function trajectories later in life. Cohort studies and recent reviews suggest that early respiratory symptoms and reduced lung function should not be viewed as isolated short-term events, since some children may follow less favorable airway-development pathways (5, 6). Identifying objective functional features during infancy and early childhood may therefore help characterize respiratory impairment more precisely, even when long-term diagnostic prediction is not yet possible.

Tidal breathing pulmonary function testing offers a feasible method for assessing lung function in infants and young children who cannot complete spirometry. The ratio of time to peak tidal expiratory flow to total expiratory time (tPTEF/tE) and the ratio of volume to peak tidal expiratory flow to total expiratory volume (VPTEF/VE) describe the relative timing and volume at which peak tidal expiratory flow is reached during quiet breathing. Lower values indicate that peak expiratory flow is reached earlier during expiration, which is compatible with an altered expiratory tidal-breathing pattern. Pediatric tidal-breathing studies have used these parameters to characterize airway obstruction or altered expiratory flow patterns in wheezing disorders, bronchiolitis-related disease, allergic airway conditions, and post-infectious bronchiolitis obliterans (7–9).

Despite these advantages, the clinical interpretation of tidal breathing indices in recurrent wheezing remains incomplete. Many studies focus on specific diseases or treatment response, while fewer studies compare recurrent wheezing with a non-wheezing clinical control group from the same testing setting. In addition, the influence of acute clinical burden, testing quality, bronchodilator exposure, and comorbid pneumonia is often difficult to separate from the wheezing phenotype itself. Therefore, retrospective data from routine clinical practice may provide useful evidence when analyzed with appropriate adjustment and sensitivity analyses.

In this study, we retrospectively analyzed infants and young children who underwent tidal breathing pulmonary function testing at a maternal and child health hospital. We compared children with recurrent wheezing with non-wheezing clinical controls, focusing on tPTEF/tE and VPTEF/VE as core expiratory tidal-breathing indices. We also examined abnormality patterns, exploratory clinical subgroups, multivariable associations, sensitivity analyses, and exploratory ROC performance. We aimed to determine whether recurrent wheezing was associated with a consistent and clinically interpretable pattern of tidal-breathing pulmonary function impairment.

## 2 Materials and methods

### 2.1 Study design and setting

This retrospective observational study was conducted in the Department of Pediatrics, Shijiazhuang Maternal and Child Health Hospital. Medical records of infants and young children who underwent tidal breathing pulmonary function testing between March 2025 and March 2026 were reviewed. The study was designed and reported in accordance with the STROBE framework for observational studies and the RECORD recommendations for studies using routinely collected clinical data (10, 11).

The study protocol was approved by the Ethics Committee of Shijiazhuang Maternal and Child Health Hospital (Approval No. 202333). Because this was a retrospective analysis of existing clinical records, the requirement for written informed consent was waived by the ethics committee.

### 2.2 Participants

Children were screened from the hospital pulmonary function database and corresponding electronic medical records. Eligible participants were infants and young children aged 3 to 36 months who had completed tidal breathing pulmonary function testing during the study period and had sufficient clinical information for group classification and outcome assessment.

The recurrent wheezing group included children with at least three physician-documented wheezing episodes before or at the index visit. Wheezing status was determined from physician records, admission notes, and respiratory diagnosis entries. This definition was consistent with recent clinical discussions of preschool wheezing, in which recurrent wheezing in young children is distinguished from a single transient wheezing episode and should be interpreted cautiously because objective asthma diagnosis is difficult in this age group (1, 2).

The non-wheezing control group included children who underwent tidal breathing pulmonary function testing during the same period but had no history of recurrent wheezing or physician-documented wheezing. These children were not considered healthy controls. Instead, they represented a non-wheezing clinical comparison group selected from the same testing setting.

Children were excluded if they were outside the study age range, had incomplete clinical or pulmonary function data, had major cardiopulmonary comorbidities or congenital airway abnormalities, or had invalid or uninterpretable tidal breathing pulmonary function tests. Invalid or uninterpretable tests referred to recordings with insufficient valid breathing cycles, marked artifacts, interrupted recording, or missing core tidal breathing indices required for analysis.

### 2.3 Data collection

Demographic and perinatal variables included age, sex, body weight, gestational age, birth weight, and premature birth. Clinical variables included passive smoking exposure, allergic history, pneumonia, bronchiolitis, suspected infant asthma, oxygen therapy, hospitalization, and bronchodilator use before testing.

Atopic risk was defined as the presence of documented personal or family allergic history. Wheezing burden was categorized within the recurrent wheezing group as 3–4 episodes or ≥5 episodes according to the recorded number of wheezing episodes. Clinical severity was categorized based on documented acute clinical status, oxygen requirement, and hospitalization status.

### 2.4 Tidal breathing pulmonary function testing

Tidal breathing pulmonary function testing was performed as part of routine clinical evaluation using an S-980A III pulmonary function meter (Sichuan Sikeda Technology Co., Ltd., China), with calibration performed according to the manufacturer's recommendations before testing. Measurements were obtained with the child in the supine position with the head maintained in a neutral midline position during quiet natural sleep or stable quiet wakefulness. An appropriately sized oronasal facemask covering both the mouth and nose was used to minimize air leakage. After a stable tidal-breathing pattern had been established, flow-volume loops were recorded. Recordings with apparent air leakage, crying or vocalization, coughing, swallowing, marked movement artifacts, interrupted breathing, or unstable respiratory patterns were excluded from analysis (7, 12).

The recorded parameters included respiratory rate (RR), tidal volume per body weight (VT/BW), inspiratory time (Ti), expiratory time (Te), the Ti/Te ratio, time to peak tidal expiratory flow as a proportion of total expiratory time (tPTEF/tE), volume to peak tidal expiratory flow as a proportion of total expiratory volume (VPTEF/VE), and peak tidal expiratory flow (PTEF). tPTEF/tE and VPTEF/VE were selected as the core tidal expiratory indices because they describe the relative timing and expired volume at which peak tidal expiratory flow occurs during quiet breathing. Lower values indicate earlier attainment of peak expiratory flow during expiration and are interpreted as an altered expiratory tidal-breathing pattern.

Low tPTEF/tE was defined as tPTEF/tE <0.28, and low VPTEF/VE was defined as VPTEF/VE <0.28. Any tidal breathing pulmonary function abnormality was defined as low tPTEF/tE or low VPTEF/VE. Both indices low was defined as simultaneous reduction of tPTEF/tE and VPTEF/VE (13).

### 2.5 Outcomes

The primary analysis compared tidal breathing pulmonary function indices between the recurrent wheezing group and the non-wheezing control group. The main outcomes were tPTEF/tE and VPTEF/VE.

Secondary analyses evaluated the proportion of children with low tPTEF/tE, low VPTEF/VE, both indices low, and any tidal breathing pulmonary function abnormality. Exploratory subgroup analyses were performed within the recurrent wheezing group according to atopic risk, wheezing burden, and clinical severity.

### 2.6 Statistical analysis

Statistical analyses were performed using IBM SPSS Statistics version 26.0 (IBM Corp., Armonk, NY, USA). Continuous variables were summarized as mean ± standard deviation, and categorical variables as number and percentage. Between-group comparisons of continuous variables were performed using independent-samples t tests; when variance heterogeneity was present, Welch correction was applied. Categorical variables were compared using Pearson chi-square tests or Fisher exact tests when expected cell counts were small. All tests were two-sided, and *P* < 0.05 was considered statistically significant.

Age was compared between children with 3–4 and ≥5 recorded wheezing episodes. The association between age and recorded wheezing episode count was assessed using Spearman's rank correlation. The between-group difference in tPTEF/tE was additionally evaluated using linear regression with age included as a covariate.

The relationship between tPTEF/tE and VPTEF/VE was assessed using Pearson's correlation coefficient, and variance inflation factors were used to evaluate multicollinearity. Both indices were entered simultaneously into a multivariable logistic regression model for recurrent wheezing status. The model was adjusted for age, male sex, premature birth, atopic risk, and bronchodilator use before testing. Both core indices were scaled per 0.01 increase, and results were reported as adjusted odds ratios with 95% confidence intervals.

Sensitivity analyses were conducted to evaluate the robustness of the primary findings. Mean differences were calculated as recurrent wheezing minus non-wheezing control. The 95% confidence intervals for mean differences were estimated using Welch standard errors with Satterthwaite degrees of freedom. Sensitivity analyses excluded premature infants, children with pneumonia, children who received oxygen therapy, and children with lower-quality but analyzable pulmonary function recordings. Additional analyses were restricted to high-quality tests only and excluded children who received bronchodilators before testing.

Receiver operating characteristic (ROC) curve analysis was performed as an exploratory analysis. The area under the curve (AUC), 95% confidence interval, optimal cutoff, sensitivity, and specificity were calculated; the optimal cutoff was identified using the Youden index. Reporting followed general diagnostic accuracy principles (14).

## 3 Results

### 3.1 Participant selection and baseline characteristics

In this study, a total of 189 medical records were screened, and 11 of them were excluded because of age ineligibility, incomplete clinical or pulmonary function data, major cardiopulmonary comorbidities or congenital airway abnormalities, or uninterpretable tidal breathing pulmonary function tests. As a result, 178 children were finally included for further review, consisting of 120 children in the recurrent wheezing group and 58 children in the non-wheezing control group (Figure 1).

**Figure 1:**
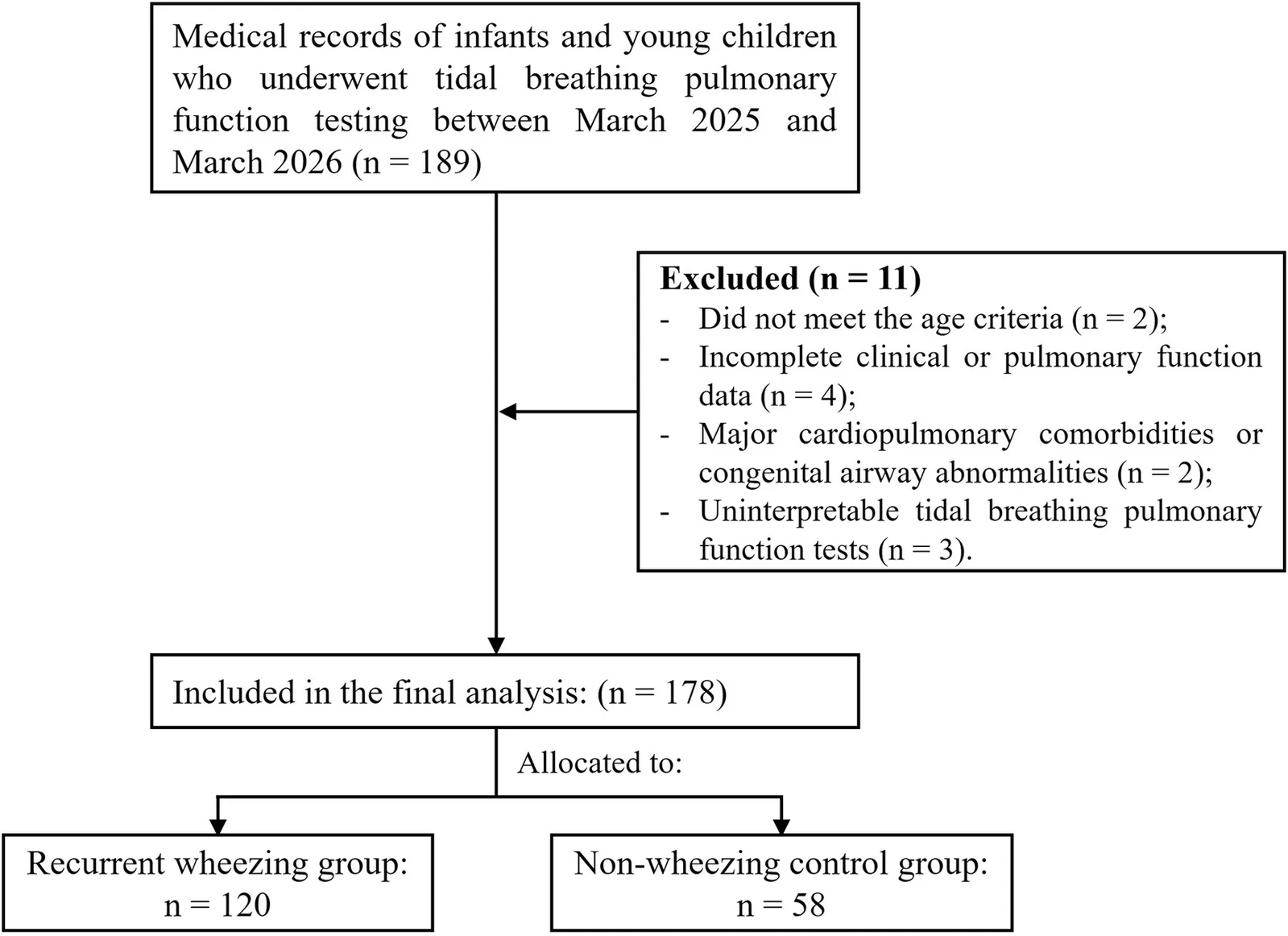
Flowchart of participant selection and group allocation.

Age, body weight, gestational age, and birth weight did not differ significantly between groups. Male sex and atopic risk were more frequent in the recurrent wheezing group. The recurrent wheezing group also had higher proportions of oxygen therapy and hospitalization, indicating greater clinical burden at or around the index assessment (Table 1).

**Table 1:** Baseline clinical characteristics of the study population.

Characteristic	Recurrent wheezing group (*n* = 120)	Non-wheezing control group (*n* = 58)	*P* value
Age, months	16.60 ± 9.07	17.19 ± 8.65	0.676
Weight, kg	10.08 ± 2.83	10.27 ± 2.64	0.656
Gestational age, weeks	38.77 ± 1.50	38.82 ± 1.28	0.828
Birth weight, g	3256.22 ± 455.32	3255.71 ± 396.94	0.994
Male sex	82 (68.3%)	28 (48.3%)	0.016
Premature birth	9 (7.5%)	3 (5.2%)	0.753
Passive smoking exposure	29 (24.2%)	10 (17.2%)	0.393
Eczema history	47 (39.2%)	17 (29.3%)	0.264
Allergic rhinitis history	20 (16.7%)	6 (10.3%)	0.372
Food allergy history	28 (23.3%)	6 (10.3%)	0.063
Family history of asthma/allergy	29 (24.2%)	9 (15.5%)	0.261
Atopic risk	70 (58.3%)	21 (36.2%)	0.009
Pneumonia	33 (27.5%)	9 (15.5%)	0.115
Bronchiolitis	31 (25.8%)	0 (0.0%)	<0.001
Suspected infant asthma	40 (33.3%)	0 (0.0%)	<0.001
Oxygen therapy	37 (30.8%)	2 (3.4%)	<0.001
Hospitalization	74 (61.7%)	18 (31.0%)	<0.001
Bronchodilator before test	14 (11.7%)	2 (3.4%)	0.094

Values are presented as mean ± SD or *n* (%). The non-wheezing control group included infants and young children who underwent tidal breathing pulmonary function testing during the same period and had no history of recurrent wheezing or physician-documented wheezing.

### 3.2 Tidal breathing pulmonary function in recurrent wheezing

The recurrent wheezing group showed a distinct pattern of tidal breathing pulmonary function impairment. The most prominent differences involved the expiratory flow timing and volume indices. Mean tPTEF/tE was lower in the recurrent wheezing group, with an absolute mean difference of approximately −0.041. VPTEF/VE showed a similar reduction, with an absolute mean difference of approximately −0.045. Both differences were statistically significant (*P* < 0.001). In contrast, Ti, Te, and Ti/Te were not significantly different between groups, suggesting that the main abnormality was concentrated in expiratory flow-related tidal breathing indices rather than in the overall timing of the respiratory cycle (Table 2 and Figure 2).

**Table 2:** Comparison of tidal breathing pulmonary function parameters between groups.

Parameter	Recurrent wheezing group (*n* = 120)	Non-wheezing control group (*n* = 58)	*P* value
RR, breaths/min	38.2 ± 4.7	36.4 ± 4.3	0.016
VT/BW, mL/kg	7.298 ± 0.786	7.641 ± 0.942	0.018
Ti, s	0.593 ± 0.118	0.589 ± 0.123	0.829
Te, s	0.800 ± 0.100	0.799 ± 0.107	0.956
Ti/Te	0.742 ± 0.123	0.734 ± 0.104	0.658
tPTEF/tE	0.277 ± 0.036	0.318 ± 0.036	<0.001
VPTEF/VE	0.289 ± 0.035	0.334 ± 0.042	<0.001
PTEF, mL/s	132.6 ± 35.5	151.3 ± 34.8	0.001

Values are presented as mean ± SD.

**Figure 2:**
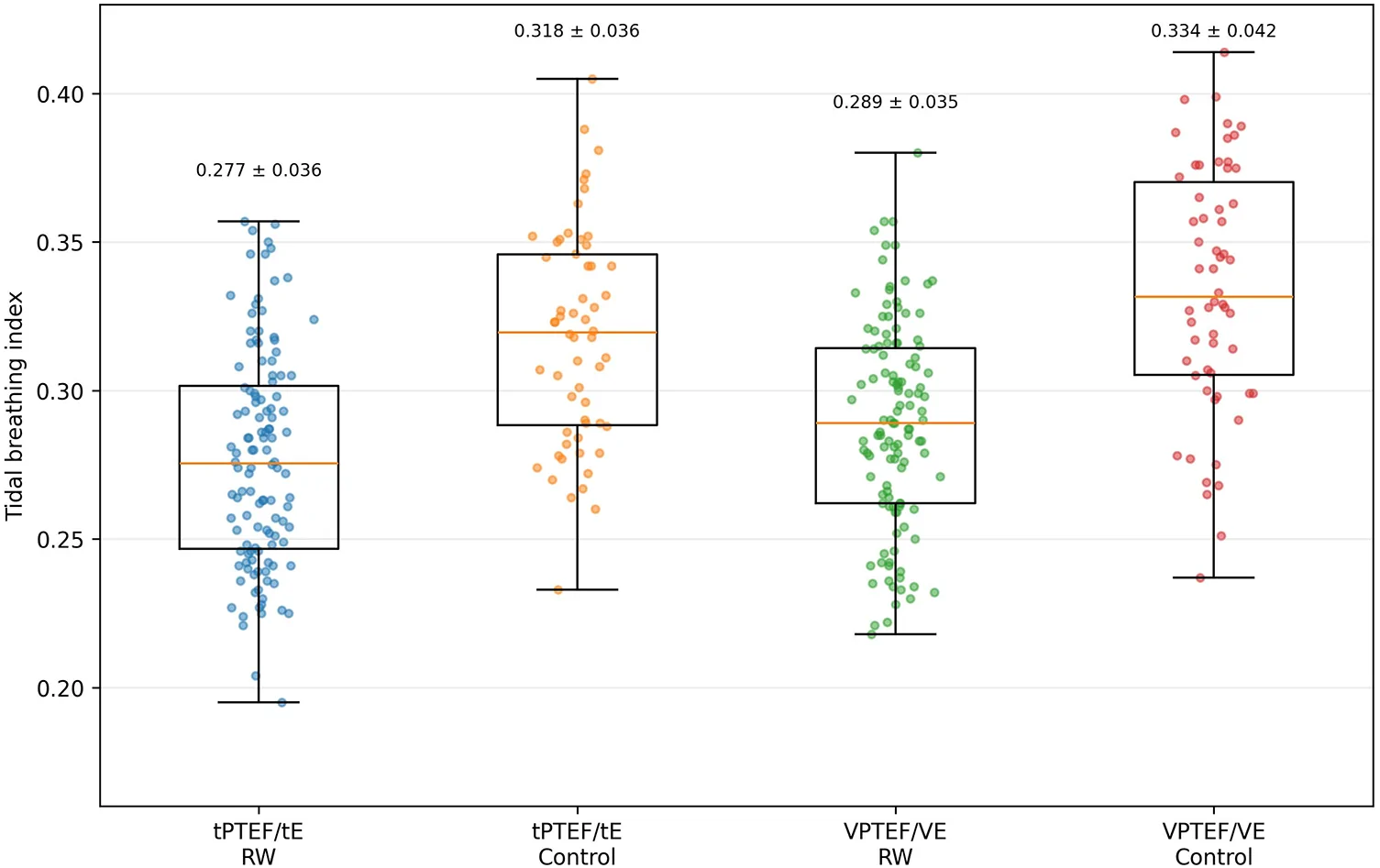
Distribution of tPTEF/tE and VPTEF/VE values in recurrent wheezing and non-wheezing control groups. RW, recurrent wheezing group; Control, non-wheezing control group.

The recurrent wheezing group also had a higher respiratory rate, lower VT/BW, and lower PTEF than the non-wheezing control group (Table 2).

### 3.3 Pattern of tidal breathing pulmonary function abnormalities

The abnormality-rate analysis reinforced the continuous-variable findings. Low tPTEF/tE, low VPTEF/VE, and simultaneous reduction of both indices were more common in the recurrent wheezing group. Any tidal breathing pulmonary function abnormality was observed in 75.8% of children with recurrent wheezing and 27.6% of non-wheezing controls. The between-group difference was therefore evident in both continuous measures and predefined abnormality patterns (Table 3 and Figure 3).

**Table 3:** Pattern of tidal breathing pulmonary function abnormalities.

Abnormality	Recurrent wheezing group (*n* = 120)	Non-wheezing control group (*n* = 58)	*P* value
Low tPTEF/tE	64 (53.3%)	11 (19.0%)	<0.001
Low VPTEF/VE	62 (51.7%)	8 (13.8%)	<0.001
Both indices low	35 (29.2%)	3 (5.2%)	<0.001
Any tidal breathing abnormality	91 (75.8%)	16 (27.6%)	<0.001

Values are presented as *n* (%). Low tPTEF/tE and low VPTEF/VE were defined as values <0.28. Any tidal breathing abnormality was defined as low tPTEF/tE and/or low VPTEF/VE.

**Figure 3:**
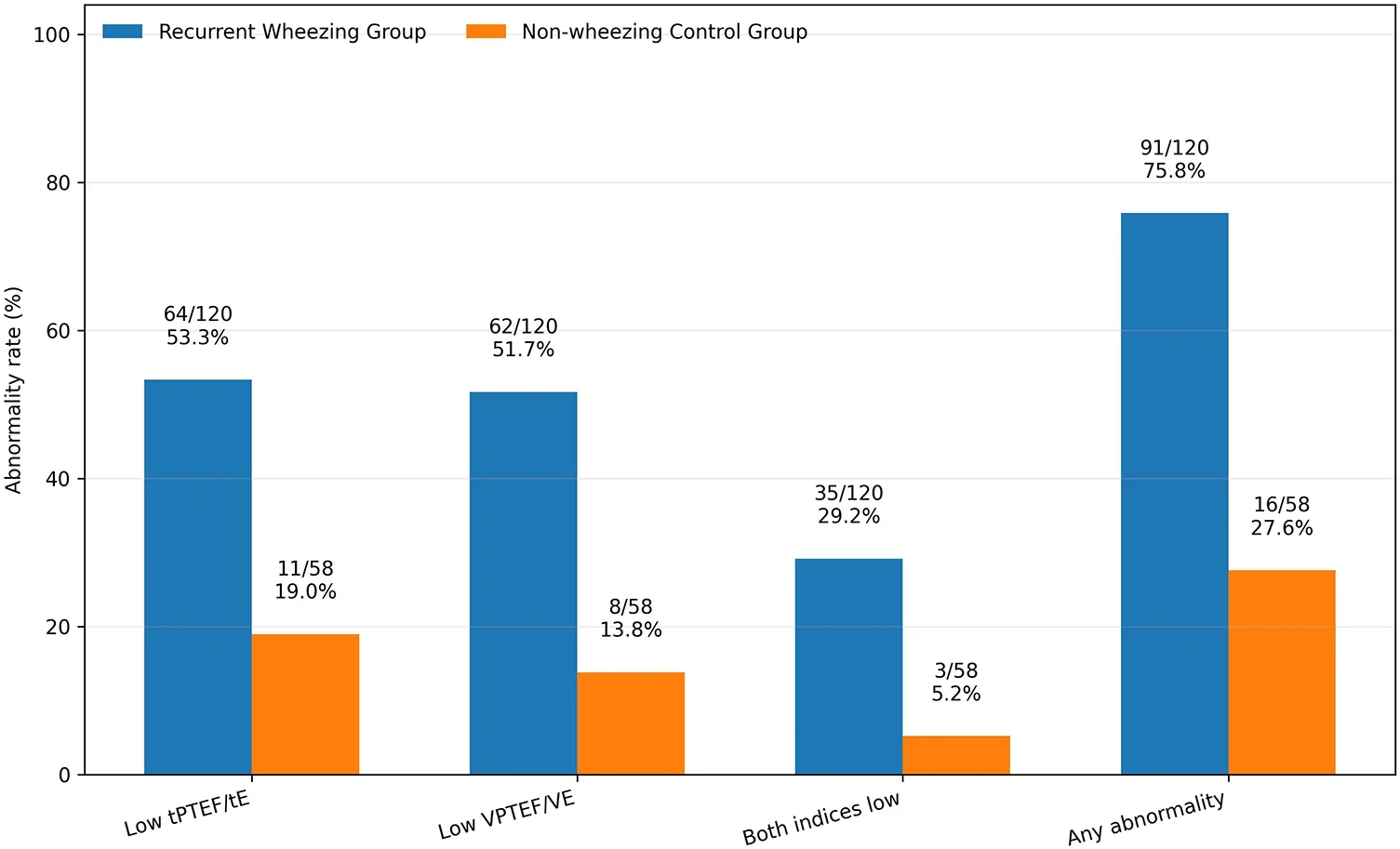
Rates of tidal breathing pulmonary function abnormalities in recurrent wheezing and non-wheezing control groups.

### 3.4 Exploratory subgroup analyses within the recurrent wheezing group

Exploratory subgroup analyses showed that atopic risk was associated with a numerically lower tPTEF/tE, but the difference did not reach statistical significance. VPTEF/VE and the overall abnormality rate were similar between children with and without atopic risk. These results suggest that atopic background alone did not fully explain the tidal breathing abnormalities observed in recurrent wheezing (Table 4).

**Table 4:** Tidal breathing pulmonary function according to atopic risk in the recurrent wheezing group.

Parameter	Atopic risk (*n* = 70)	Non-atopic risk (*n* = 50)	*P* value
tPTEF/tE	0.272 ± 0.037	0.284 ± 0.035	0.096
VPTEF/VE	0.290 ± 0.034	0.287 ± 0.036	0.704
RR, breaths/min	38.2 ± 4.7	38.2 ± 4.8	0.943
VT/BW, mL/kg	7.383 ± 0.803	7.178 ± 0.752	0.156
PTEF, mL/s	129.6 ± 36.6	136.7 ± 33.8	0.273
Any tidal breathing abnormality	56 (80.0%)	35 (70.0%)	0.280

Values are presented as mean ± SD or *n* (%).

When children with recurrent wheezing were stratified by wheezing burden, those with ≥5 episodes had lower tPTEF/tE than those with 3–4 episodes. Age was comparable between the two wheezing-burden subgroups (16.14 ± 9.62 vs 16.86 ± 8.80 months; *P* = 0.687), and the recorded number of wheezing episodes was not significantly correlated with age (Spearman *ρ*=-0.099, *P* = 0.284). After adjustment for age, the difference in tPTEF/tE remained significant (adjusted difference, −0.0158; 95% CI, −0.0294 to −0.0022; *P* = 0.023). VPTEF/VE and the overall abnormality rate did not differ significantly between the two groups. The subgroup analysis was exploratory (Table 5).

**Table 5:** Tidal breathing pulmonary function according to wheezing burden in the recurrent wheezing group.

Parameter	≥5 episodes (*n* = 43)	3–4 episodes (*n* = 77)	*P* value
tPTEF/tE	0.267 ± 0.036	0.283 ± 0.035	0.023
VPTEF/VE	0.284 ± 0.036	0.291 ± 0.034	0.292
RR, breaths/min	39.0 ± 4.8	37.8 ± 4.7	0.188
VT/BW, mL/kg	7.142 ± 0.780	7.384 ± 0.780	0.106
PTEF, mL/s	134.0 ± 34.5	131.8 ± 36.2	0.745
Any tidal breathing abnormality	35 (81.4%)	56 (72.7%)	0.375
Age, months	16.14 ± 9.62	16.86 ± 8.80	0.687

Values are presented as mean ± SD or *n* (%). Age was comparable between wheezing-burden groups (*P* = 0.687), and the recorded number of wheezing episodes was not correlated with age (Spearman *ρ*=-0.099, *P* = 0.284). After adjustment for age, the ≥5-episode group retained a lower tPTEF/tE (adjusted difference, −0.0158; 95% CI, −0.0294 to −0.0022; *P* = 0.023).

Clinical severity analysis showed that children in the higher-severity subgroup had a higher respiratory rate and numerically lower tPTEF/tE and VPTEF/VE. The difference in any tidal breathing pulmonary function abnormality showed a trend but did not reach statistical significance. These findings indicate that clinical severity may influence tidal breathing patterns, but the evidence was not strong enough to support an independent severity-based conclusion (Table 6).

**Table 6:** Tidal breathing pulmonary function according to clinical severity in the recurrent wheezing group.

Parameter	Higher severity (*n* = 67)	Lower severity (*n* = 53)	*P* value
tPTEF/tE	0.272 ± 0.035	0.284 ± 0.038	0.073
VPTEF/VE	0.284 ± 0.036	0.294 ± 0.033	0.123
RR, breaths/min	39.0 ± 4.6	37.2 ± 4.7	0.037
VT/BW, mL/kg	7.196 ± 0.780	7.426 ± 0.781	0.110
PTEF, mL/s	134.2 ± 32.7	130.5 ± 39.0	0.584
Any tidal breathing abnormality	55 (82.1%)	36 (67.9%)	0.088

Values are presented as mean ± SD or *n* (%).

### 3.5 Multivariable logistic regression

tPTEF/tE and VPTEF/VE showed a weak positive correlation (Pearson r = 0.279, *P* < 0.001). Variance inflation factors were 1.12 and 1.11, respectively, indicating no important multicollinearity. Both indices were therefore entered simultaneously into a combined multivariable logistic regression model. After adjustment for age, male sex, premature birth, atopic risk, and bronchodilator use before testing, each 0.01 increase in tPTEF/tE was associated with lower odds of recurrent wheezing (adjusted OR, 0.746; 95% CI, 0.656–0.848; *P* < 0.001). A similar independent association was observed for VPTEF/VE (adjusted OR, 0.746; 95% CI, 0.663–0.840; *P* < 0.001) (Table 7).

**Table 7:** Multivariable logistic regression analysis for recurrent wheezing status including both core tidal breathing indices.

Model	Predictor	Adjusted OR	95% CI	*P* value
Combined model	tPTEF/tE, per 0.01 increase	0.746	0.656–0.848	<0.001
	VPTEF/VE, per 0.01 increase	0.746	0.663–0.840	<0.001
	Age, months	0.983	0.935–1.033	0.492
	Male sex	2.285	0.956–5.463	0.063
	Premature birth	3.953	0.595–26.253	0.155
	Atopic risk	1.935	0.816–4.584	0.134
	Bronchodilator before test	6.377	0.784–51.892	0.083

The core tidal breathing parameters were scaled per 0.01 increase. tPTEF/tE and VPTEF/VE showed a weak positive correlation (Pearson *r* = 0.279, *P* < 0.001). Variance inflation factors were 1.12 and 1.11, respectively, indicating no important multicollinearity and supporting simultaneous inclusion of both indices in the same model. The model was adjusted for age, male sex, premature birth, atopic risk, and bronchodilator use before testing.

### 3.6 Sensitivity analyses

Sensitivity analyses showed that the main findings were robust across clinically relevant exclusions. After excluding premature infants, children with pneumonia, lower-quality but analyzable tests, and children who received bronchodilators before testing, the mean differences in tPTEF/tE and VPTEF/VE remained negative and the 95% confidence intervals did not cross zero. Restricting the analysis to high-quality tests only produced the same direction of effect (Table 8 and Figure 4).

**Table 8:** Sensitivity analyses of mean differences in core tidal breathing indices.

Sensitivity analysis	Parameter	Mean difference	95% CI	*n* (Recurrent wheezing group/non-wheezing control group)
All cases	tPTEF/tE	−0.041	−0.052 to −0.029	120/58
	VPTEF/VE	−0.045	−0.058 to −0.033	120/58
Excluding premature infants	tPTEF/tE	−0.039	−0.051 to −0.028	111/55
	VPTEF/VE	−0.046	−0.060 to −0.033	111/55
Excluding pneumonia	tPTEF/tE	−0.045	−0.057 to −0.032	87/49
	VPTEF/VE	−0.042	−0.056 to −0.027	87/49
Excluding lower-quality but analyzable tests	tPTEF/tE	−0.038	−0.050 to −0.027	117/52
	VPTEF/VE	−0.049	−0.062 to −0.037	117/52
High-quality tests only	tPTEF/tE	−0.037	−0.051 to −0.024	91/40
	VPTEF/VE	−0.054	−0.068 to −0.040	91/40
Excluding bronchodilator before test	tPTEF/tE	−0.041	−0.053 to −0.029	106/56
	VPTEF/VE	−0.046	−0.058 to −0.033	106/56
Excluding premature + pneumonia + lower-quality but analyzable tests	tPTEF/tE	−0.040	−0.053 to −0.026	78/40
	VPTEF/VE	−0.048	−0.063 to −0.033	78/40
Excluding oxygen therapy	tPTEF/tE	−0.043	−0.056 to −0.030	83/56
	VPTEF/VE	−0.044	−0.057 to −0.031	83/56

Lower-quality but analyzable tests referred to recordings that met the minimum criteria for primary analysis but did not meet the stricter high-quality criteria used in the sensitivity analysis.

**Figure 4:**
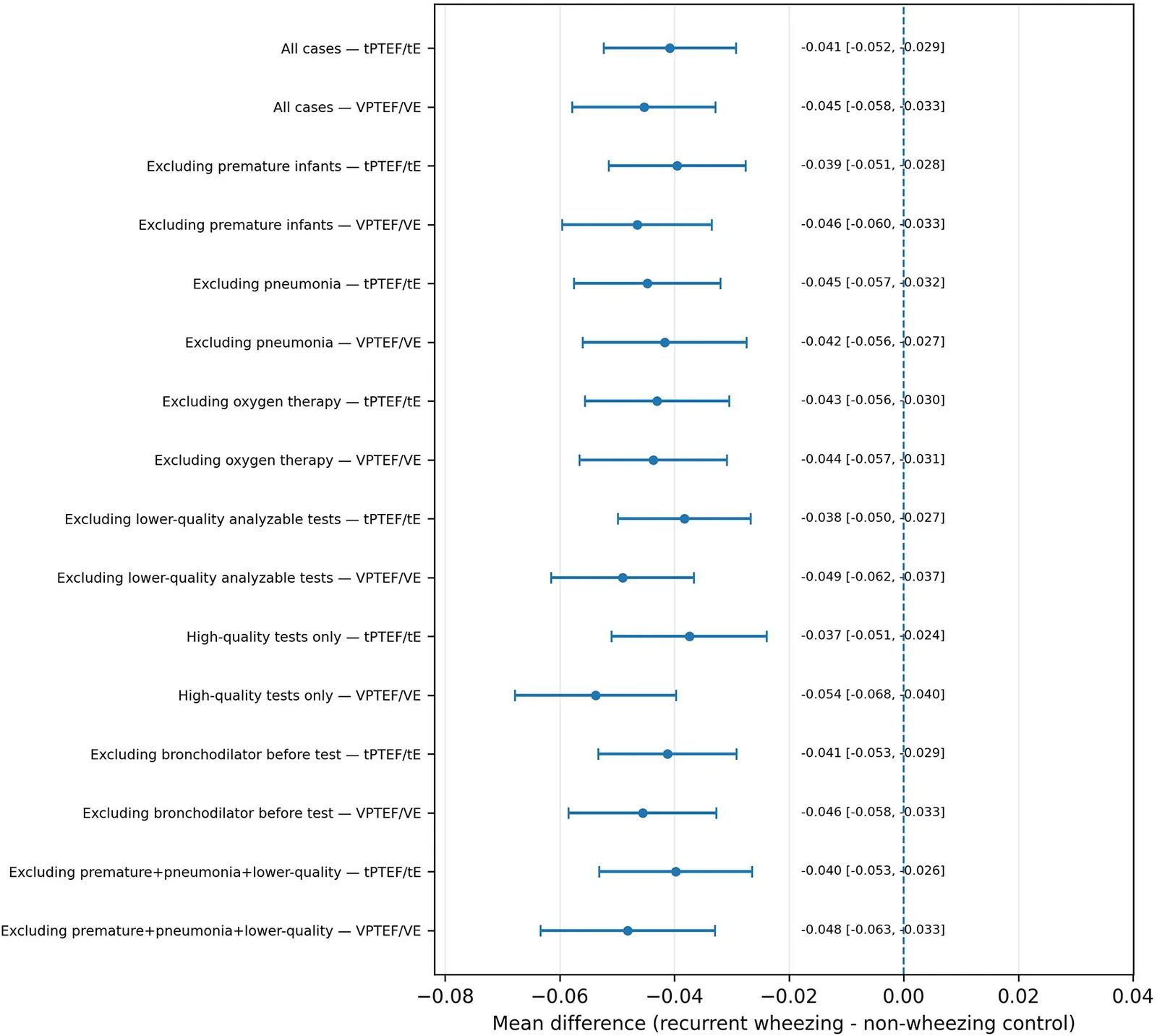
Sensitivity analysis forest plot for mean differences in tPTEF/tE and VPTEF/VE.

An additional sensitivity analysis excluded children who received oxygen therapy. The mean differences remained −0.043 for tPTEF/tE and −0.044 for VPTEF/VE, similar in direction and magnitude to the primary analysis.

### 3.7 Exploratory ROC analysis

The exploratory ROC analysis showed moderate discriminatory ability for both core tidal breathing indices. The area under the curve was 0.779 for tPTEF/tE and 0.792 for VPTEF/VE. The optimal cutoffs identified by the Youden index provided moderate sensitivity and specificity rather than diagnostic-level performance. These findings suggest that tPTEF/tE and VPTEF/VE may provide supportive objective information for evaluating recurrent wheezing in young children, but they should not be interpreted as standalone diagnostic tests (Figure 5).

**Figure 5:**
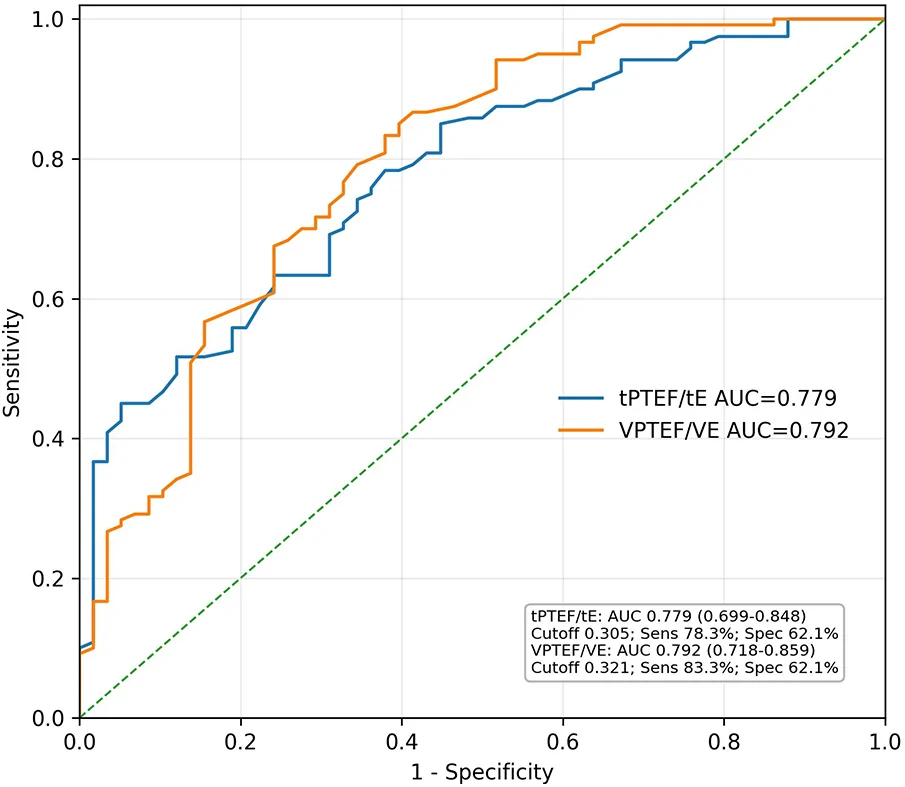
ROC curves for tPTEF/tE and VPTEF/VE in distinguishing recurrent wheezing from controls.

## 4 Discussion

In this retrospective study of 178 infants and young children, recurrent wheezing was associated with lower tPTEF/tE and VPTEF/VE compared with non-wheezing clinical controls. The observed pattern was not a global reduction in all tidal breathing parameters. Instead, the main differences were concentrated in indices related to the timing and volume proportion of peak tidal expiratory flow, whereas Ti, Te, and Ti/Te were similar between groups. This suggests that the pulmonary function abnormality in recurrent wheezing was more closely related to expiratory flow pattern than to overall respiratory cycle timing.

This finding is clinically meaningful because tPTEF/tE and VPTEF/VE are commonly interpreted as tidal-breathing indicators of altered expiratory flow in young children. Reduced values indicate that peak expiratory flow is reached earlier during expiration, a pattern that may be compatible with altered small or peripheral airway mechanics during tidal breathing (7, 15). In infants and young children, these findings should also be considered within the context of rapid airway and lung development. Longitudinal birth-cohort data indicate that early-life respiratory exposures can influence lung-function development from infancy through the preschool years (16). In this context, our results extend previous pediatric tidal-breathing observations by showing that this expiratory pattern can be detected in a recurrent-wheezing population using routine clinical data and remains evident after multiple sensitivity analyses. Importantly, the difference between groups was also reflected in abnormality-rate analysis, as children with recurrent wheezing had a higher proportion of low tPTEF/tE, low VPTEF/VE, and any tidal-breathing abnormality. This supports the view that recurrent wheezing in early childhood is accompanied by a broader shift in tidal expiratory breathing pattern rather than only a small difference in mean values. Similar use of tidal-breathing parameters has been reported in pediatric airway diseases, but our study specifically emphasizes recurrent wheezing in a real-world infant and toddler cohort (8, 9).

These results align with established knowledge that preschool children who have recurrent wheezing form a heterogeneous group but are frequently characterized by impaired airway function. The 2024 European Respiratory Society statement highlights the need for better objective markers beyond symptom-based phenotypes in preschool wheezing, because clinical labels may not fully capture underlying mechanisms or future risk (2). The Global Initiative for Asthma also emphasizes that asthma diagnosis in children aged 5 years or younger requires careful assessment (1). Early-life wheeze is known to be associated with later respiratory morbidity from previous studies; however, the magnitude of the association differs between wheezing phenotypes, viral aetiology and atopic background (5, 17). Specifically, both respiratory syncytial virus and rhinovirus-related bronchiolitis have been associated with subsequent recurrent wheezing and asthma risk, with the latter having a stronger association in certain cohorts (18). Although this study did not assess viral etiology or long-term asthma, it still provides objective tidal breathing evidence in such clinical settings.

Longitudinal lung-function trajectory studies provide another framework for interpreting the present findings. Children with persistently low or worsening lung-function trajectories may contribute disproportionately to later airflow obstruction and chronic respiratory morbidity (6, 19). Although our study was retrospective and cross-sectional, lower tPTEF/tE and VPTEF/VE in children with recurrent wheezing suggest that tidal-breathing testing may help describe early functional differences before conventional spirometry becomes feasible. These indices should be interpreted as measures of altered tidal expiratory flow pattern rather than direct diagnostic markers of asthma. Tidal-breathing measurements are influenced by respiratory state, sleep or wakefulness, mask fit, and technical quality. Bains and colleagues showed that infant tidal flow-volume parameters vary by arousal state, supporting careful standardization of testing conditions (20). More broadly, pulmonary-function results should be interpreted in conjunction with technical quality and clinical context (21).

Sensitivity analyses yielded similar estimates after excluding premature infants, pneumonia cases, lower-quality but analyzable tests, bronchodilator exposure before testing, and oxygen therapy. The consistency of the findings after exclusion of oxygen-treated children suggests that greater acute illness burden did not fully account for the observed group differences, although residual confounding remains possible. Exploratory ROC analysis showed moderate discriminatory ability for both tPTEF/tE and VPTEF/VE; the observed sensitivity and specificity support their use as supplementary physiologic measures rather than standalone classifiers. This interpretation is consistent with general diagnostic-accuracy reporting principles (22).

Atopic risk was more common in the recurrent wheezing group, which is consistent with previous studies showing that allergic constitution contributes to preschool wheeze and later asthma susceptibility (23, 24). However, within the recurrent wheezing group, atopic risk was not strongly associated with worse tidal-breathing indices, suggesting that atopic background alone did not account for the observed functional differences. In contrast, children with ≥5 wheezing episodes had lower tPTEF/tE than those with 3–4 episodes, a finding that is biologically plausible in the context of recurrent airway narrowing and airway hyperresponsiveness (16, 25, 26). Age was comparable between wheezing-burden subgroups, the recorded number of episodes was not significantly correlated with age, and the tPTEF/tE difference remained significant after age adjustment. VPTEF/VE and the overall abnormality rate did not differ significantly between wheezing-burden subgroups; therefore, the wheezing-burden finding should be interpreted as exploratory.

This study has several strengths. It focused on infants and young children, a population in which conventional spirometry is difficult and objective functional assessment is limited. Use of a non-wheezing clinical comparison group from the same testing setting and sensitivity analyses across clinically relevant exclusions addressed several sources of heterogeneity, including pneumonia, oxygen therapy, bronchodilator exposure, and pulmonary-function test quality. The analyses considered continuous indices, predefined abnormality patterns, multivariable associations, subgroup findings, and exploratory ROC performance, providing complementary perspectives on tidal-breathing pulmonary function in recurrent wheezing.

Several limitations should be acknowledged. First, the retrospective single-center design limits causal interpretation and external generalizability. The proportion of oxygen therapy and hospitalization was higher in the recurrent wheezing group, and although sensitivity analyses supported the main findings, severity-associated unmeasured confounding may remain. Second, the non-wheezing control group was a clinical comparison group rather than a healthy community control group. This maintained a similar testing context across groups, but the results should not be interpreted as differences between children with recurrent wheezing and healthy children. Third, the study did not include longitudinal follow-up for subsequent asthma diagnosis, lung-function trajectory, or treatment response; therefore, the findings cannot establish whether reduced tPTEF/tE or VPTEF/VE predicts persistent asthma. Future prospective studies should combine repeated tidal-breathing testing with symptom trajectories, inflammatory markers, and later spirometry when children become old enough to cooperate. Finally, tidal-breathing indices are sensitive to technical and behavioral factors. Although measurements followed the routine laboratory protocol and invalid recordings were excluded, arousal state could not be prospectively standardized in this retrospective dataset, and some residual variability related to quiet sleep or wakefulness and mask fit may remain. Future studies should use prespecified acquisition states and detailed quality-control procedures (15, 20).

Taken together, the findings suggest that tidal breathing pulmonary function testing may provide useful objective information in infants and young children with recurrent wheezing, particularly when spirometry is not feasible. Reduced tPTEF/tE and VPTEF/VE may help characterize altered expiratory tidal-breathing mechanics, while wheezing burden may provide additional clinical context. Future prospective studies integrating repeated tidal-breathing measurements with longitudinal clinical outcomes are needed to clarify their prognostic relevance.

## 5 Conclusion

Infants and young children with recurrent wheezing showed lower tPTEF/tE and VPTEF/VE than non-wheezing clinical controls, indicating a consistent pattern of altered tidal expiratory breathing mechanics. The findings remained stable across sensitivity analyses excluding clinically relevant factors, including oxygen therapy, pneumonia, premature birth, bronchodilator exposure, and lower-quality but analyzable tests. Exploratory subgroup analyses suggested that wheezing burden and clinical severity may be related to some tidal-breathing indices, but these findings should be interpreted cautiously.

Tidal breathing pulmonary function testing may provide supportive objective information for evaluating recurrent wheezing in infants and young children who cannot perform spirometry. However, tPTEF/tE and VPTEF/VE should not be used as standalone diagnostic markers. Larger prospective studies with standardized testing protocols and longitudinal follow-up are needed to clarify their role in early risk stratification and respiratory outcome assessment.

## Data Availability

The original contributions presented in the study are included in the article/Supplementary Material, further inquiries can be directed to the corresponding author.
